# Supplementary material for: Progranulin mediates immune evasion of pancreatic ductal adenocarcinoma through regulation of MHCI expression
Source: Nat Commun. 2022 Jan 10;13:156. doi: 10.1038/s41467-021-27088-9 (PMC8748938; doi:10.1038/s41467-021-27088-9)
Supplement: Supplementary file 1 — Supplementary Information [file 41467_2021_27088_MOESM1_ESM.pdf]

Supplementary Figure 1

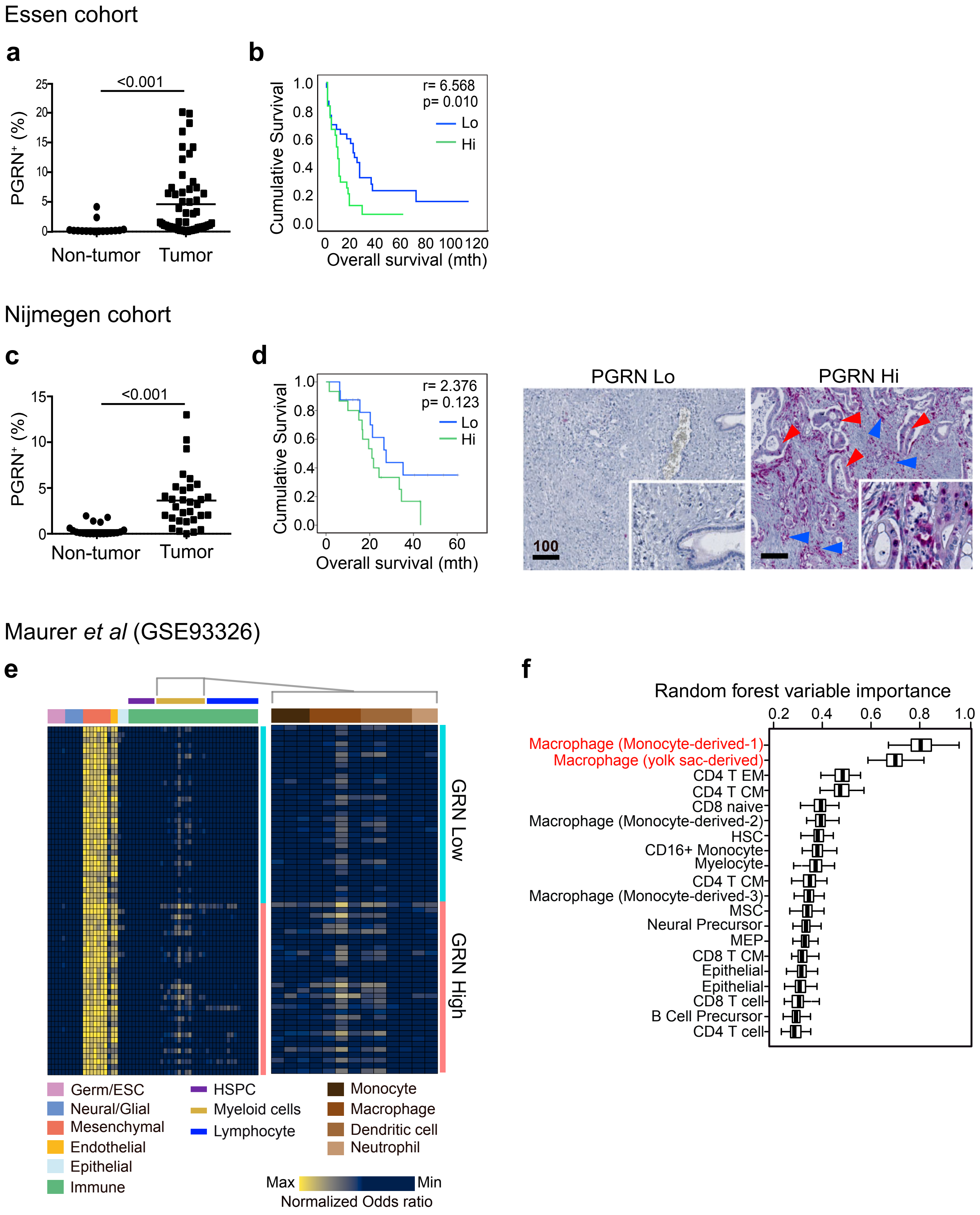

Supplementary Figure 1
(a,b) Essen cohort. (a) PGRN+ cells in tumor (n=54) and adjacent non-tumor (n=16) tissues of human PDAC was assessed by IHC staining and quantified by Definiens. Two-tailed Mann-Whitney test. Median ± SD is shown. (b) Kaplan-Meier overall survival plot according to PGRN expression level. Patients were segregated into low (n=30) and high (n=24) expression groups with median of number of PGRN+ cells as cutoff. Log rank test. (c,d) Nijmegen cohort (n=31). (c) PGRN expression levels in tumor (n=31) and adjacent non-tumor (n=21) tissues of human PDAC were assessed by IHC staining and quantified by Definiens. Two-tailed Mann-Whitney test. Median ± SD is shown. (d) Kaplan-Meier overall survival plots according to PGRN expression level. Patients (n= 31) were segregated into low (n=16) and high (n=15) expression groups with median of positive cells as cutoff. Log rank test. Right panel shows representative IHC staining of high- and low- PGRN expressing patient specimens. Right panel: PGRN expression in both tumor and stromal compartments of PDAC. Red arrowheads indicate PGRN+ tumor cells; blue arrowheads indicate PGRN+ stromal cells. (e,f) Maurer *et al* dataset (GSE93326, n=65). (e) Cell type deconvolution of 43 different cell types of transcriptomes derived from *GRN*-high (*n* =32) and *GRN*-low (*n* =32) stroma samples, indicating enrichment of myeloid cells in *GRN*-high stroma. Data represents normalized odds ratios comparing number of enriched cell type-specific genes with random enrichment for each sample (rows) and cell type (columns). HSPC, Hematopoietic stem and progenitor cells. (f) Box plots represent top 20 variable importance computed by 100-fold random forest for each cell type identified in (e), and ordered by descending importance in predicting *GRN* high and low stroma samples. Macrophages represents the cell type showing highest degree of importance in predicting *GRN* high stroma. Boxplots are drawn with boxes representing the interquartile range (IQR), a line across the box indicating the median, and whiskers indicating 1.5 x IQR. Scale bar unit: μm

Supplementary Figure 2

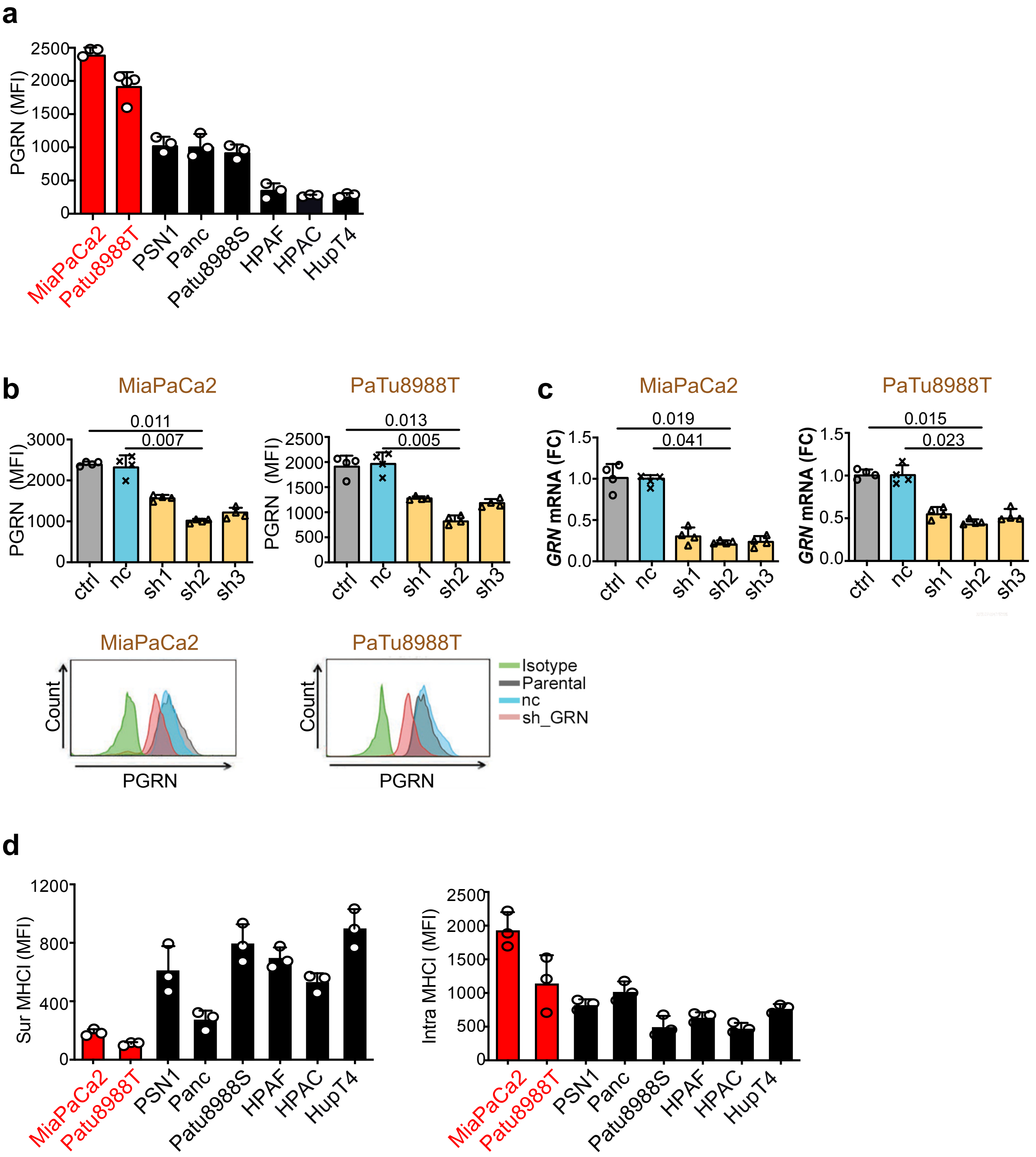

Supplementary Figure 2
(a) Expression level of intracellular PGRN in human commercial PDAC cell lines was assessed by flow cytometric analysis. PatuT: n=4; All others: n=3 independent experiments. (b,c) *GRN* suppression by shRNA plasmid transfection in PDAC cell lines MiaPaCa2 and Patu8988T. PGRN protein (PGRN) and gene (*GRN*) levels measured by (b) flow cytometry and (c) qRT-PCR, respectively. *GRN* shRNA2 (sh2) consistently demonstrated the highest efficiency of suppression and was therefore selected for subsequent functional evaluation as "shGRN". n=4 independent experiments. One-way ANOVA, Kruskal-Wallis test. (d) Expression level of surface and intracellular MHCI (HLA-A/B/C) in human commercial PDAC cell lines was assessed by flow cytometric analysis. n=3 independent experiments. FC: fold change. ctrl: parental PDAC cells; nc: shRNA scrambled control; sh1-3; *GRN* shRNA1-3. MFI: mean fluorescence intensity. Mean + SD is shown.

Supplementary Figure 3

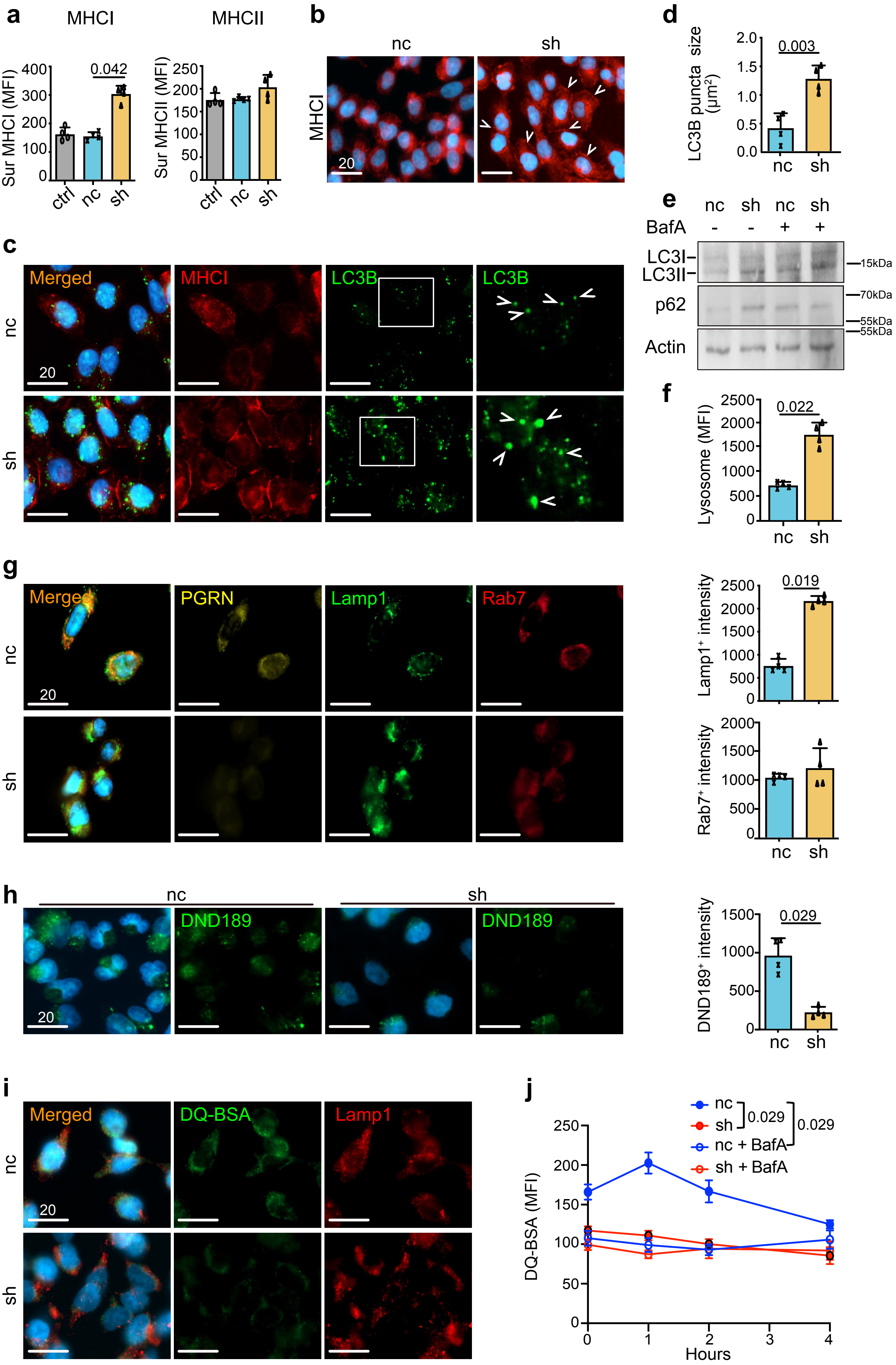

Supplementary Figure 3

(a) Surface MHCI (HLA-A/B/C) and MHCII (HLA-DR) expression on human PDAC cell line Patu8988T upon *GRN* suppression was assessed by flow cytometry. n=4 independent experiments. One-way ANOVA, Kruskal-Wallis test. MFI: mean fluorescence intensity. (b) IF staining of MHCI marker HLA-A/B/C reveals augmented surface MHCI expression on Patu8988T upon *GRN* suppression. White arrowheads indicate the membraneous staining of MHCI. (n=4 independent experiments). Representative images are shown. (c,d) IF staining of MHCI (red) and LC3B (green) in Patu8988T cells upon *GRN* suppression. (d) Average size of LC3B puncta of 50 cells of each treatment was measured by ZEN software. n=4 independent experiments. Two-tailed Mann-Whitney test. (e) Western blot showing LC3B (LC3I, II), p62/SQSTM1 (p62) and actin of Patu8988T cells upon *GRN* suppression treated with or without V-ATPase inhibitor Balfinomycin A (BafA, 100nM, 24h). (n=4 independent experiments). Representative images are shown. Two-tailed Mann-Whitney test. (f) Lysosome content in Patu8988T upon *GRN* suppression was assessed by staining with Cytopainter LysoGreen indicator and measured by flow cytometry. n=4 independent experiments. Two-tailed Mann-Whitney test. (g) IF staining of PGRN, lysosome marker Lamp1 and late endosome marker Rab7 in Patu8988T upon *GRN* suppression. Right panel: Average intensity of Lamp1 and Rab7 signals per cell was measured by HALO software. n=4 independent experiments. Two-tailed Mann-Whitney test. (h) IF staining of LysoSensor DND-189 in Patu8988T cells upon *GRN* suppression. Right panel: Average intensity of DND189 signal per cell was measured by HALO software. n=4 independent experiments. Two-tailed Mann-Whitney test. (i) IF images showing the dequenched DA-BSA (green) and Lamp1 (red) in Patu8988T cells upon *GRN* suppression. (j) Flow cytometric quantification of the dequenched DQ-BSA signal in Patu8988T cells upon *GRN* suppression, with or without prior treatment with BafA (100nM, 24h), after different chasing time. n=4 independent experiments. Two-tailed Mann-Whitney test. ctrl: parental PDAC cells; nc: shRNA scrambled control; sh; *GRN* shRNA. Mean + or ± SD is shown. Scale bar unit: µm

Supplementary Figure 4

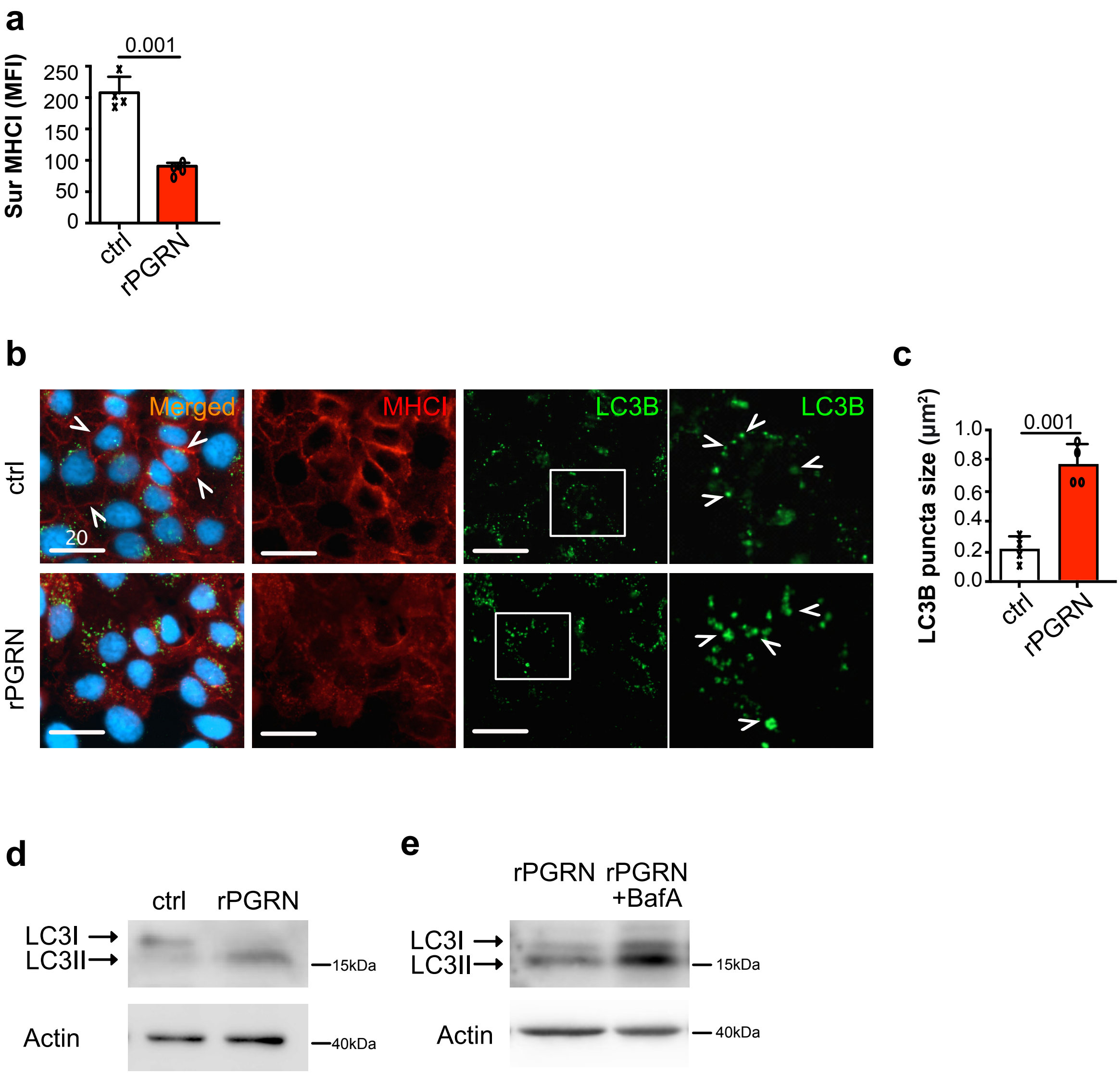

Supplementary Figure 4

HupT4 cells were treated with or without 0.4 ng/mL recombinant PGRN (rPGRN) for 1 day. (a) Surface MHCI (HLA-A/B/C) was assessed by flow cytometry. n=4 independent experiments. Two-tailed Mann-Whitney test. (b,c) IF staining of MHCI (red) and LC3B (green) in HupT4 cells upon rPGRN stimulation. White arrows on the left image indicates membranous staining of MHCI; while the arrows on the right image indicates LC3 puncta. (n=4 independent experiments). Representative images are shown. (c) Average size of LC3B puncta of 50 cells of each treatment was measured by ZEN software. n=4 independent experiments. Two-tailed Mann-Whitney test. (d) Western blot showing LC3B-II level of HupT4 cells with or without rPGRN (0.4ng/ml, 24h). (n=3 independent experiments). Representative images are shown. (e) Western blot showing LC3B-II level of HupT4 treated with rPGRN (0.4ng/ml, 24h) with or without co-treatment with BafA (100nM, 24h). (n=3 independent experiments). Representative images are shown. Mean + SD is shown. Scale bar unit: μm

Supplementary Figure 5

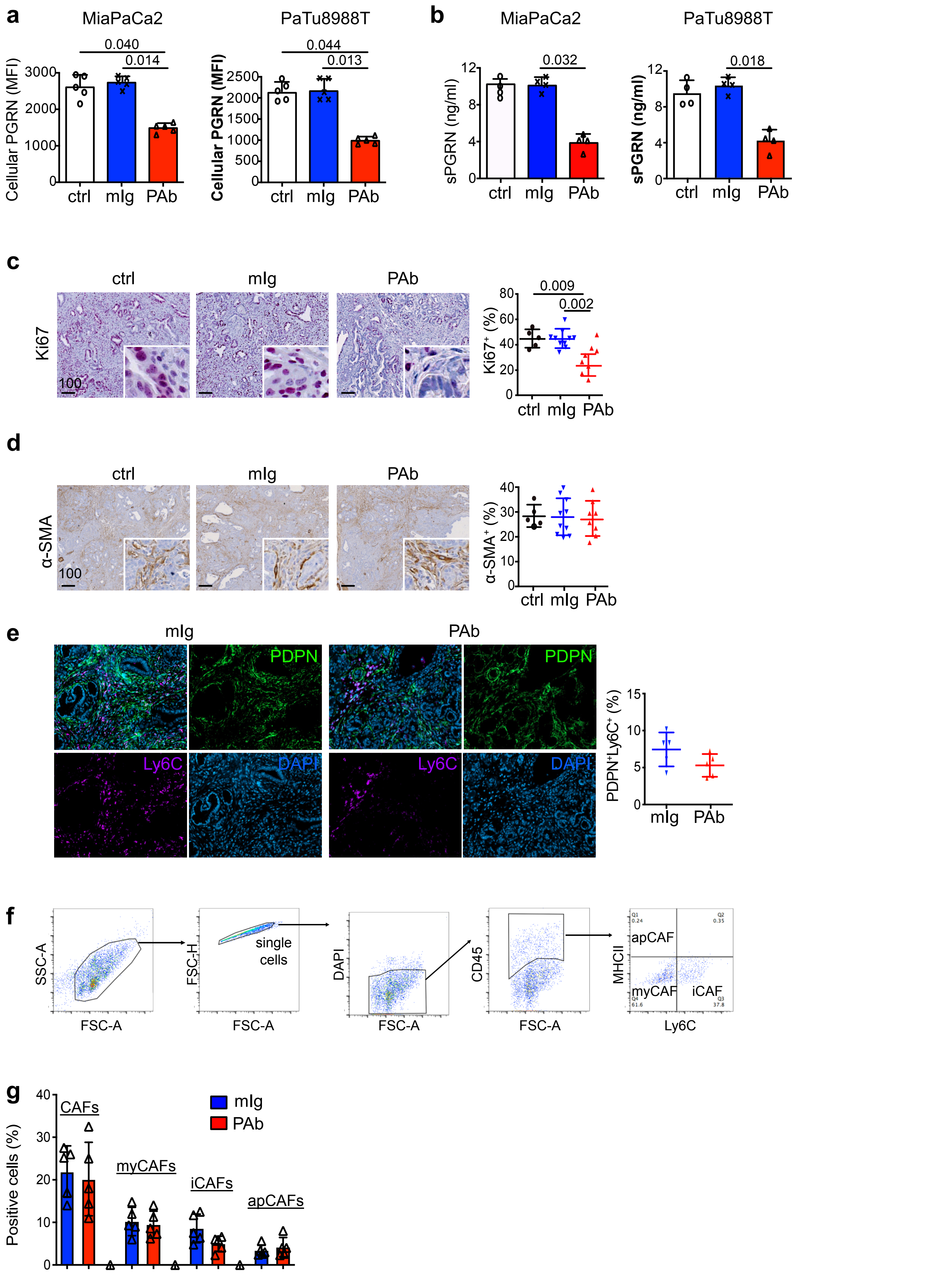

Supplementary Figure 5

MiaPaCa2 and Patu8988T cells were s treated with PGRN Ab (PAb) or mIg (100ug/ml) for 24h. Medium was then changed and cells were cultured for 2 more days. (a) Cellular PGRN level was assessed by flow cytometry (n=5 independent experiments), while (b) soluble PGRN (sPGRN) level was measured by ELISA (n=4 culture supernatents collected from cultured cells in 4 independent experiments). One-way ANOVA, Kruskal-Wallis test. MFI: Mean Fluorescent Intensity. (c,d) IHC staining of (c) Ki67 and (d) myofibroblast marker α-sma in *CKP* tumors treated with or without PGRN Ab (PAb) or mIg. Right panel shows the percentage of positive cells in the tumors (n=5). ctrl: n=5 animals; mIg: n=10 animals; PGRN Ab (PAb): n=8 animals. One-way ANOVA, Kruskal-Wallis test. (e) mIF staining of PDPN (green) and Ly6C (purple) in *CKP* tumors treated with PGRN Ab or mIg. Right panel shows the percentage of $PDPN^+Ly6C^+$ iCAFs in the tumors (n=5 animals). Two-tailed Mann-Whitney test. (f) Gating strategy for flow cytometry analysis on different CAF populations. (g) Flow cytometric analysis on the quantity of PDPN+ CAFs, $PDPN^+Ly6C^+MHCII^-$ iCAFs, $PDPN^+Ly6C^-MHCII^-$ myCAFs, and $PDPN^+Ly6C^-MHCII^+$ apCAFs in *CKP* tumors treated with PGRN Ab or mIg (50mg/kg) (n=5 animals). Two-tailed Mann-Whitney test. Mean + or $\pm$ SD is shown.

Supplementary Figure 6

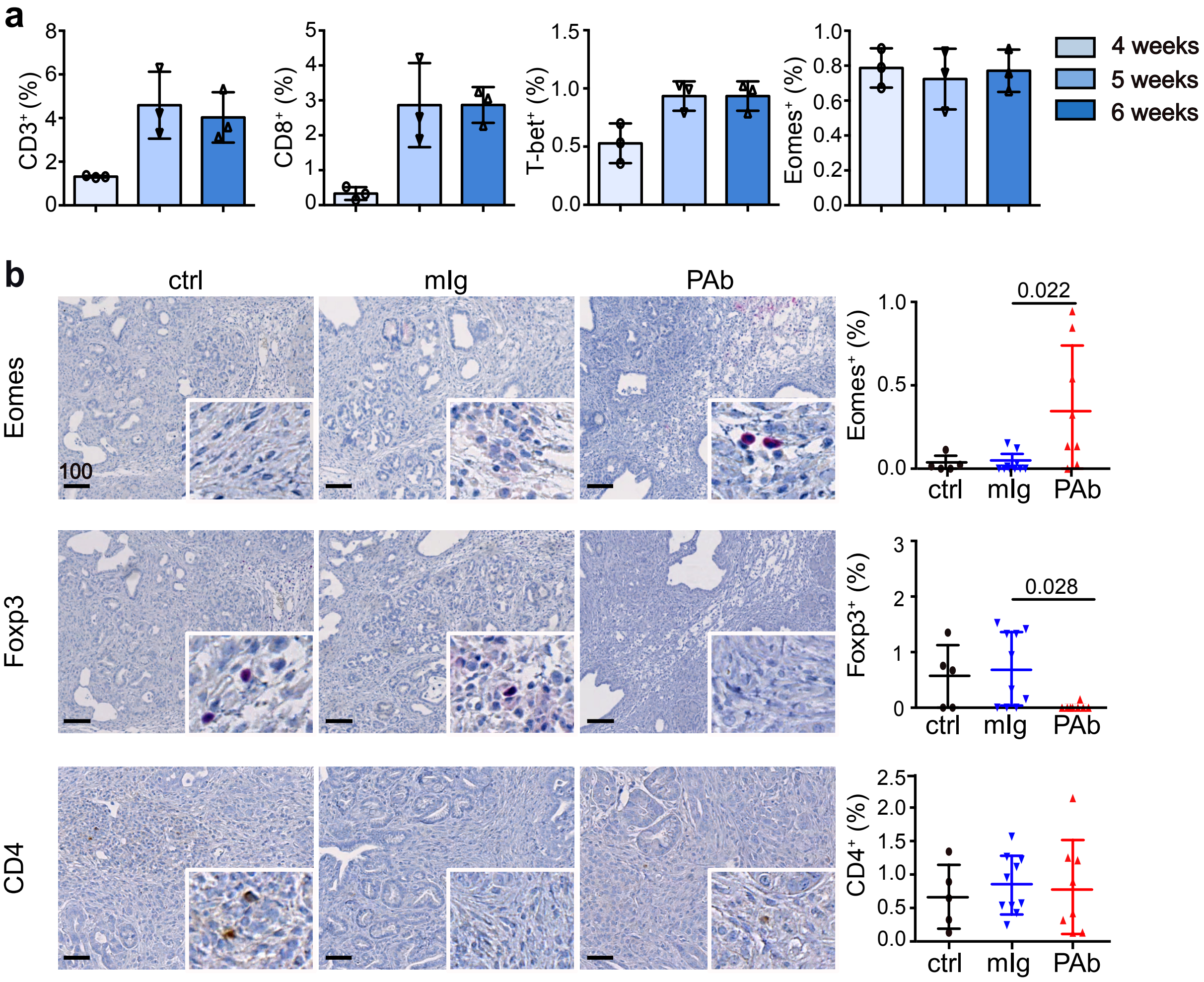

Supplementary Figure 6

(a) Quantification of T cell markers CD3, CD8, T-bet and Eomes in *CKP* tumors during early PDAC development (n=3 animals from each time point). One-way ANOVA, Kruskal-Wallis test. Mean ± SD is shown. (b) IHC of CD4, Eomes and Foxp3 in *CKP* tumors treated with or without PGRN Ab or mIg. ctrl: n=5 animals; mIg: n=10 animals; PGRN Ab (PAb): n=8 animals.The right panel shows the percentage of positive cells in the whole tumorous tissues. ctrl: n=5 animals; mIg: n=10 animals; PGRN Ab: n=8 animals. One-way ANOVA, Kruskal-Wallis test. Mean ± SD is shown. Scale bar unit: μm

Supplementary Figure 7

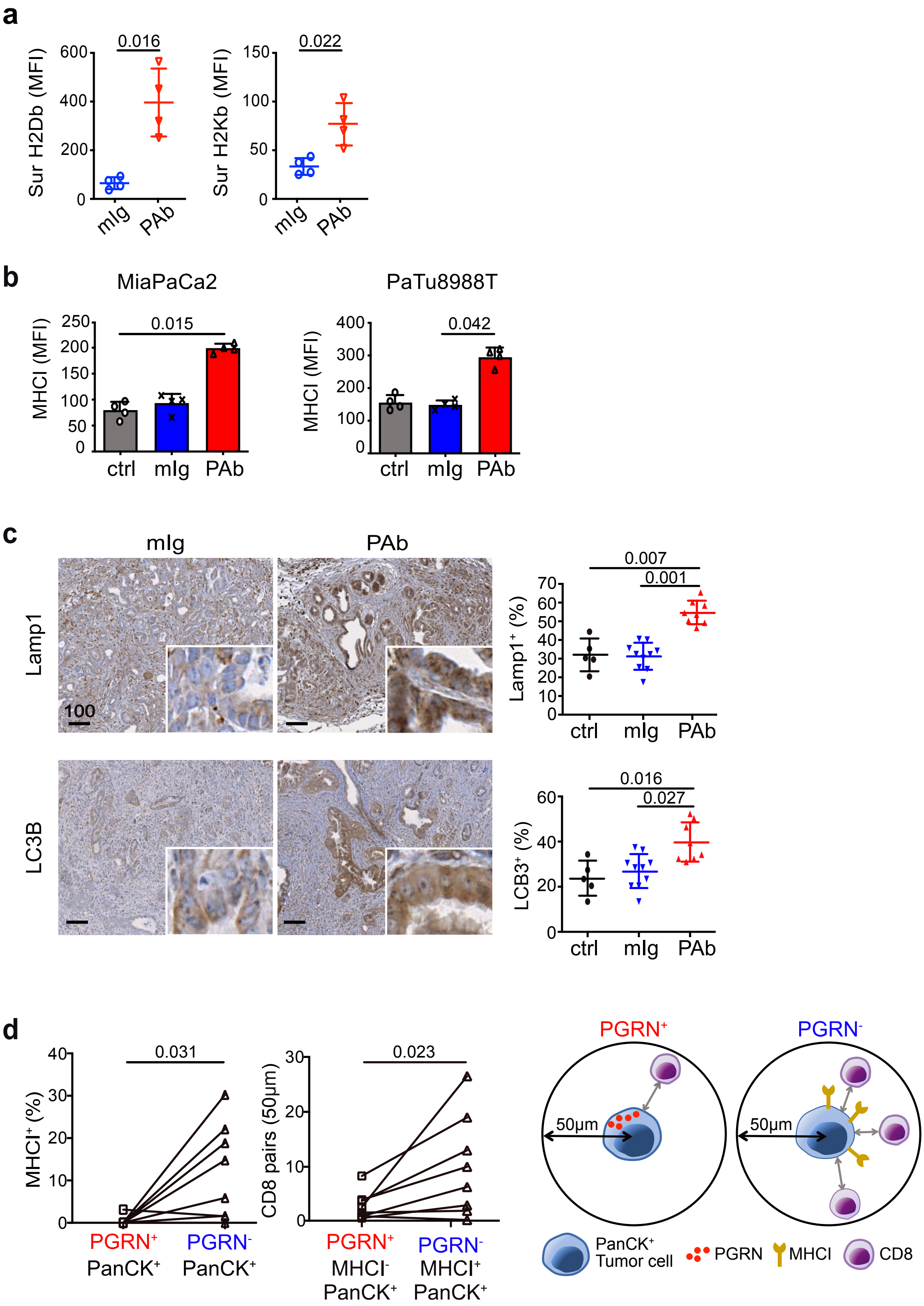

Supplementary Figure 7

(a) Surface MHCI molecules H-2Db and H-2Kb expression on $EpCAM^+$ tumor cells freshly dissociated from *CKP* tumors treated with PGRN Ab or mIg (50mg/kg) were assessed by flow cytometry (n=4 animals). Two-tailed Mann-Whitney test. Mean ± SD is shown. (b) Surface MHCI (HLA-A/B/C) on human PDAC cell lines MiaPaCa2 and Patu8988T upon treatment with PGRN Ab or mIg (100 ug/ml) was assessed by flow cytometry (n=4 independent experiments). One-way ANOVA, Kruskal-Wallis test. Mean + SD is shown. (c) IHC staining of lysosome marker Lamp1 and autophagosome marker LC3B in *CKP* tumors treated with mIg or PGRN Ab. The right panels show the percentage of $Lamp1^+$ and $LC3B^+$ cells in the whole tumorous tissues of *CKP* mice treated with or without PGRN Ab and mIg. ctrl: n=5 animals; mIg: n=10 animals; PGRN Ab (PAb): n=8 animals. One-way ANOVA, Kruskal-Wallis test. Mean ± SD is shown. (d) Automated computational analysis showing the percentage of $MHCI^+$ cells in $PGRN^+PanCK^+$ and PGRN-$PanCK^+$ populations (left panel), and the number of $CD8^+$ cells in proximity (<50μm radical distance) of $PGRN^+MHCI^-$ or $PGRN^-MHCI^+$ $PanCK^+$ tumor cells in anti-PGRN Ab-treated *CKP* tumors (n=8 animals) (right panel). Two-tailed Mann-Whitney test. Scheme depicts the differential MHCI expression and interaction of tumor and CD8 cells depending on tumor-expressed PGRN. Scale bar unit: μm

Supplementary Figure 8

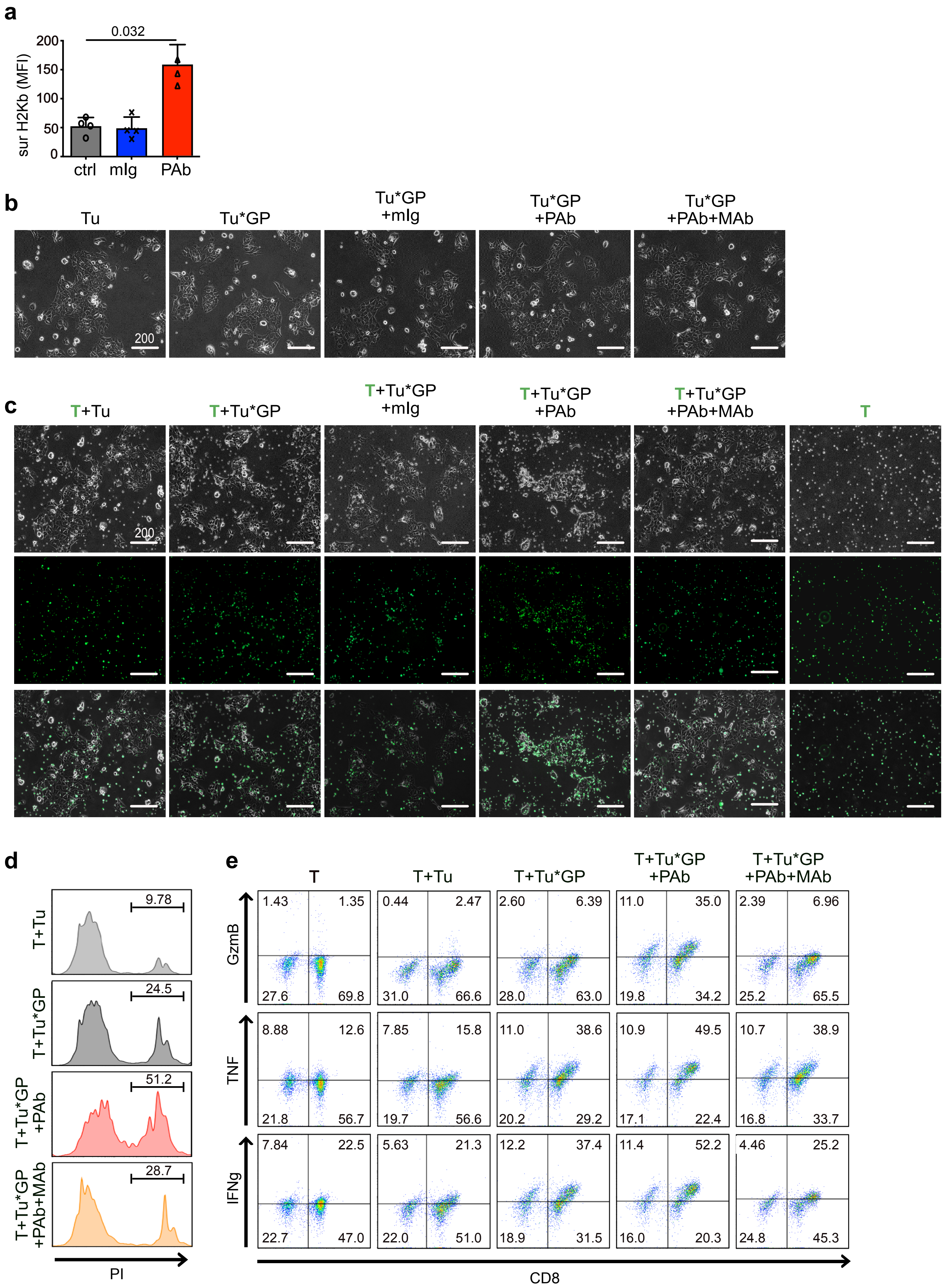

Supplementary Figure 8
(a) Surface expression of MHCI marker H-2Kb on GP82 cells treated with or without PGRN Ab or mIg (100ug/ml) was assessed by flow cytometry (n=4 indepentdent experiments). One-way ANOVA, Kruskal-Wallis test. Mean + SD is shown. (b) Phase-contrast images of GP82 cells cultured with different treatments for 2 days. (c) Phase-contrast, fluorescence, and overlay images of GP82 cells and LCMV-gp33-reactive T cells (CFSE-labeled, green) after 2 days of co-culture. When anti-MHCI (H-2Db) neutralizing antibody (MAb) was included in the treatment, MAb was added 1h after PGRN Ab treatment. T cells were then added 1h after MAb treatment. Tu: Tumor cells (GP82 cells); GP: LCMV-gp33-induced; PAb: PGRN antibody (100ug/ml); MAb: MHCI (H-2Db) neutralizing antibody (100ug/ml). (d) Representative histograms showing cytotoxicity level of GP82 cells with or without LCMV-gp33 expression, anti-PGRN antibody, and anti-MHCI neutralizing antibody, upon co-culture with LCMV-gp33-reactive T cells. Percentages of PI^+ cells are indicated. (e) Representative scatter plots showing the percentage of cells that are positive for both CD8 and cytotoxic markers granzyme B (GzmB), TNF, and IFNg.

Supplementary Figure 9

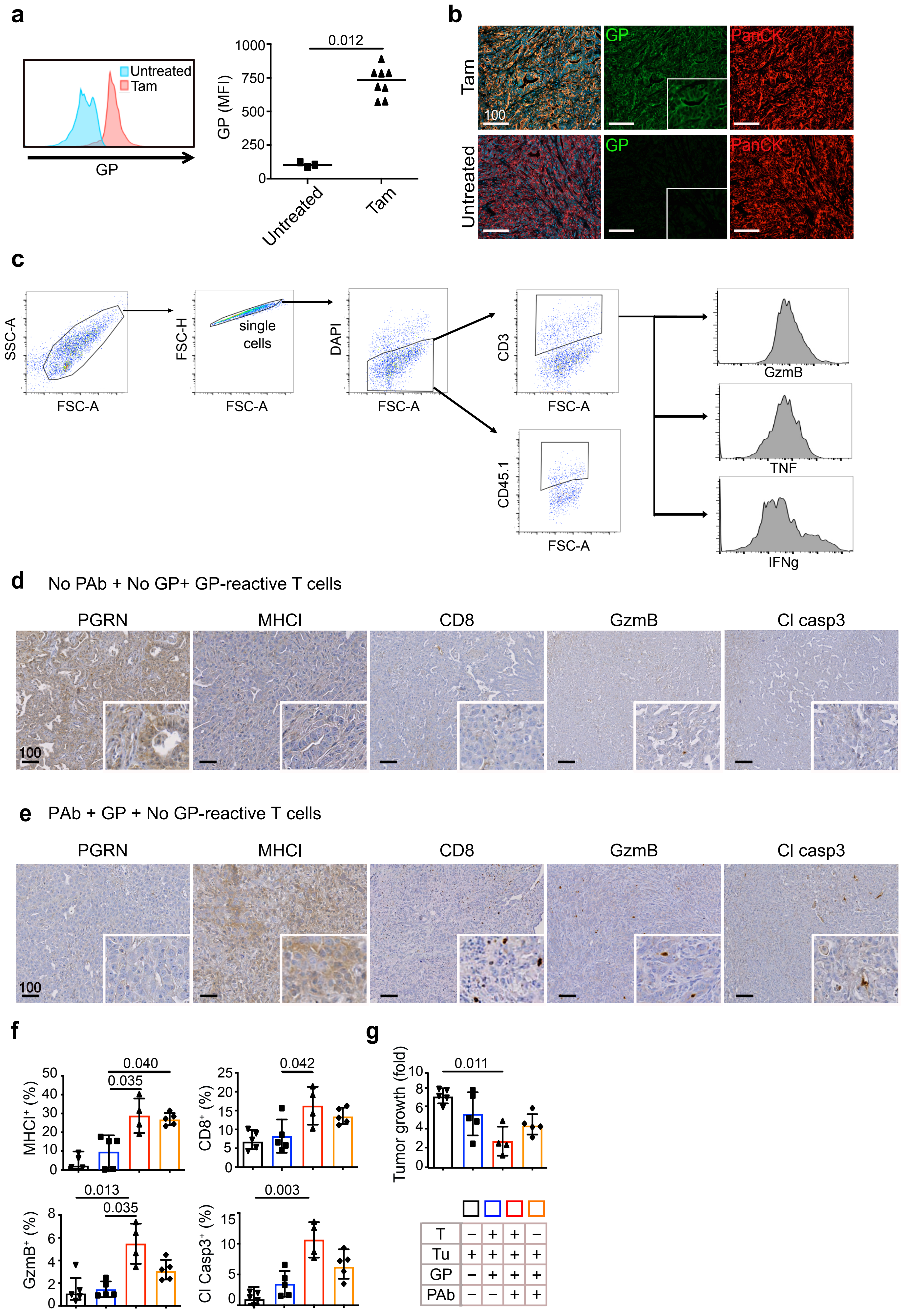

T	−	+	+	−
Tu	+	+	+	+
GP	−	+	+	+
PAb	−	−	+	+

Supplementary Figure 9

(a) Tumors dissected from GP82 orthotopic models that are treated with or without tamoxifen (Tam, 75mg/kg) were digested into disaggregated cells. Cells were stained for LCMV-gp33 (GP) expression and analyzed by flow cytometry. Untreated: n=3; Tam: n=8. Two-tailed Mann-Whitney test. MFI: Mean Fluorescent Intensity. (b) IF staining showing LCMV-gp33 (GP) expression in PanCK$^+$ tumor cells in GP82 orthotopic models that were treated with or without tamoxifen (Tam, 75mg/kg). (n=3 untreated or Tam-treated GP82 tumors). Representative images are shown. (c) Gating strategy for flow cytometric analysis on the T cell quantification and characterization in dissociated tumors. (d, e) IHC staining of PGRN, MHCI, CD8, GranzymB (GzmB), and cleaved casp3 (Cl casp3) in tumor (d) without tamoxifen and PGRN Ab treatment, but with LCMV-gp33-reactive T cell injection (n=3 animals); and (e) with tamoxifen and PGRN Ab treatment, but without LCMV-gp33-reactive T cell injection (n=4 animals). Representative images are shown. (f) Percentage of respective positive cells in the whole tumors quantified by Definiens. (g) Tumor growth was assessed by ultrasound imaging and presented as fold change in tumor volume before and after PGRN Ab or mIg treatment started. (f, g) Tumor only: n=5; Tumor with LCMV-gp33 (GP) and LCMV-gp33-reactive T cell: n=5; Tumor with LCMV-gp33 (GP) and LCMV-gp33-reactive T cell and PGRN antibody: n=4; Tumor with LCMV-gp33 (GP) and PGRN antibody: n=4. One-way ANOVA, Kruskal-Wallis test. Mean ± SD is shown. Scale bar unit: μm

Supplementary Figure 10

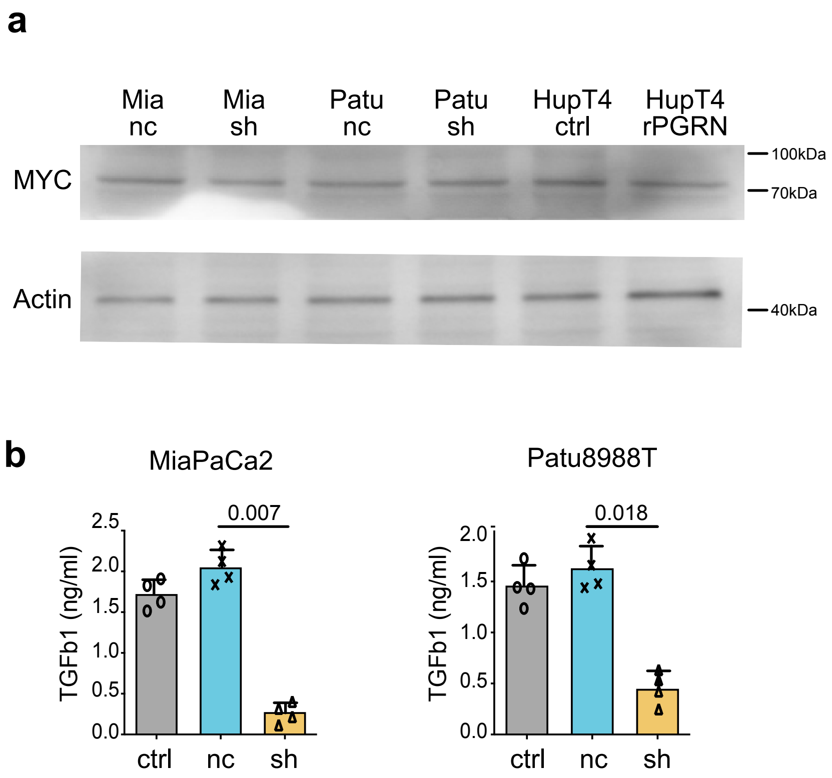

Supplementary Figure 10
(a) Western blot showing MYC and actin in MiaPaCa2 (Mia) and Patu8988T (Patu) cells with or without *GRN* suppression (nc: scrable control; sh: shGRN); or HupT4 cells treated with or without 0.4 ng/ml rPGRN for 24h. (n=3 independent experiments). Representative images are shown. (b) TGFb1 level in culture supernatants of control (nc) and shGRN transfectants of MiaPaCa2 and PatuT was measured by ELISA (n=4). One-way ANOVA, Kruskal-Wallis test. Mean + SD is shown.

Supplementary Figure 11

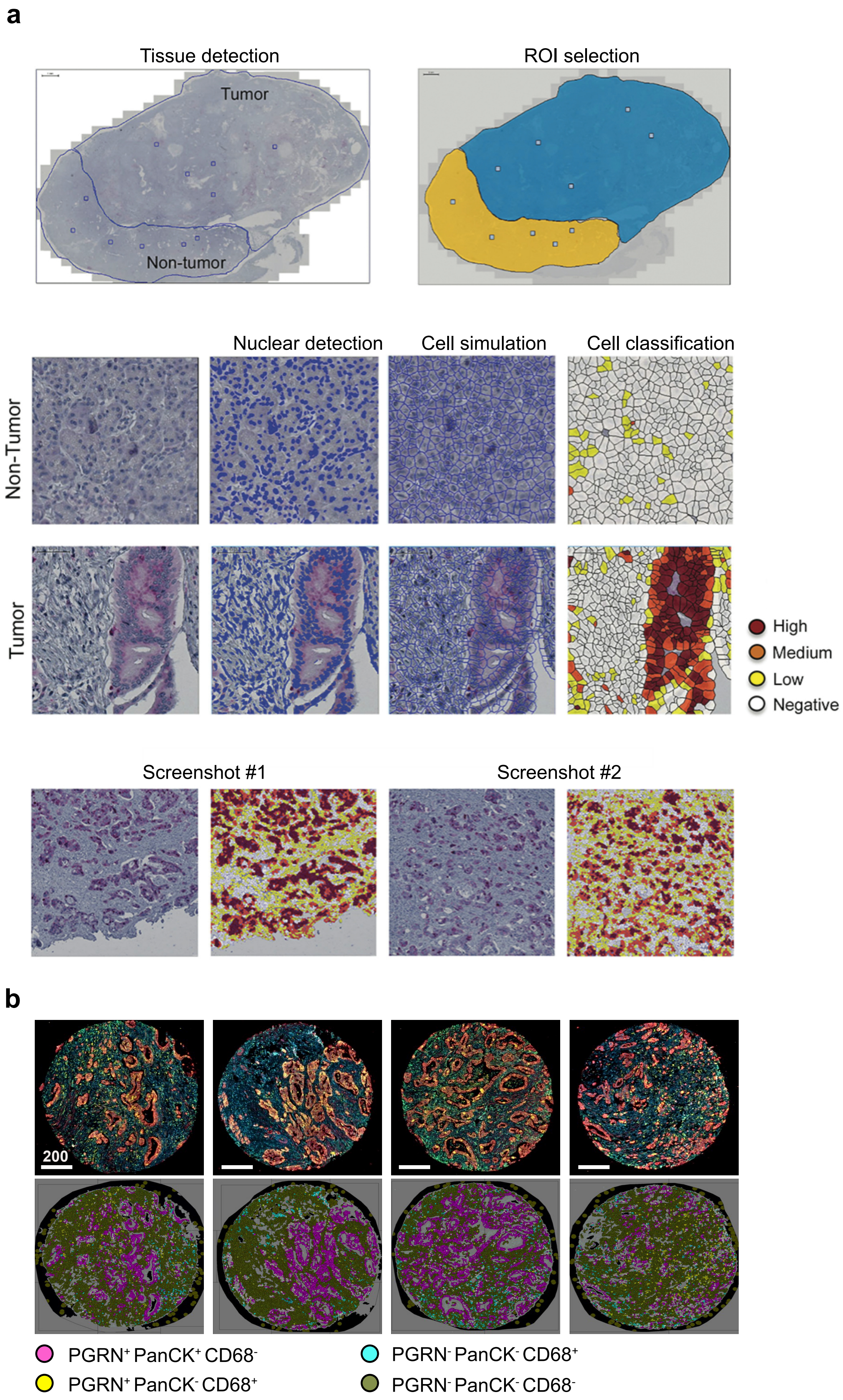

Supplementary Figure 11

Quantification of IHC staining by Definiens. (a) Tumor and non-tumor pancreas tissue were defined. Regions of interest (ROIs) were selected for defining nucleus and cells, for accurate assessment of cell number of each slide. Cell classification was performed to categorized different signal intensity. Screenshot #1 and #2 as examples to illustrate the accuracy of the software. (n=54 for Essen cohort, n=31 for Nijmegen cohort). Representative images are shown. (b) Quantification of mIF staining in TMA. Examples showing quantification of cells co-expressing PGRN and/or PanCK and/or CD68. (n=71). Representative images are shown.

Supplementary Figure 12

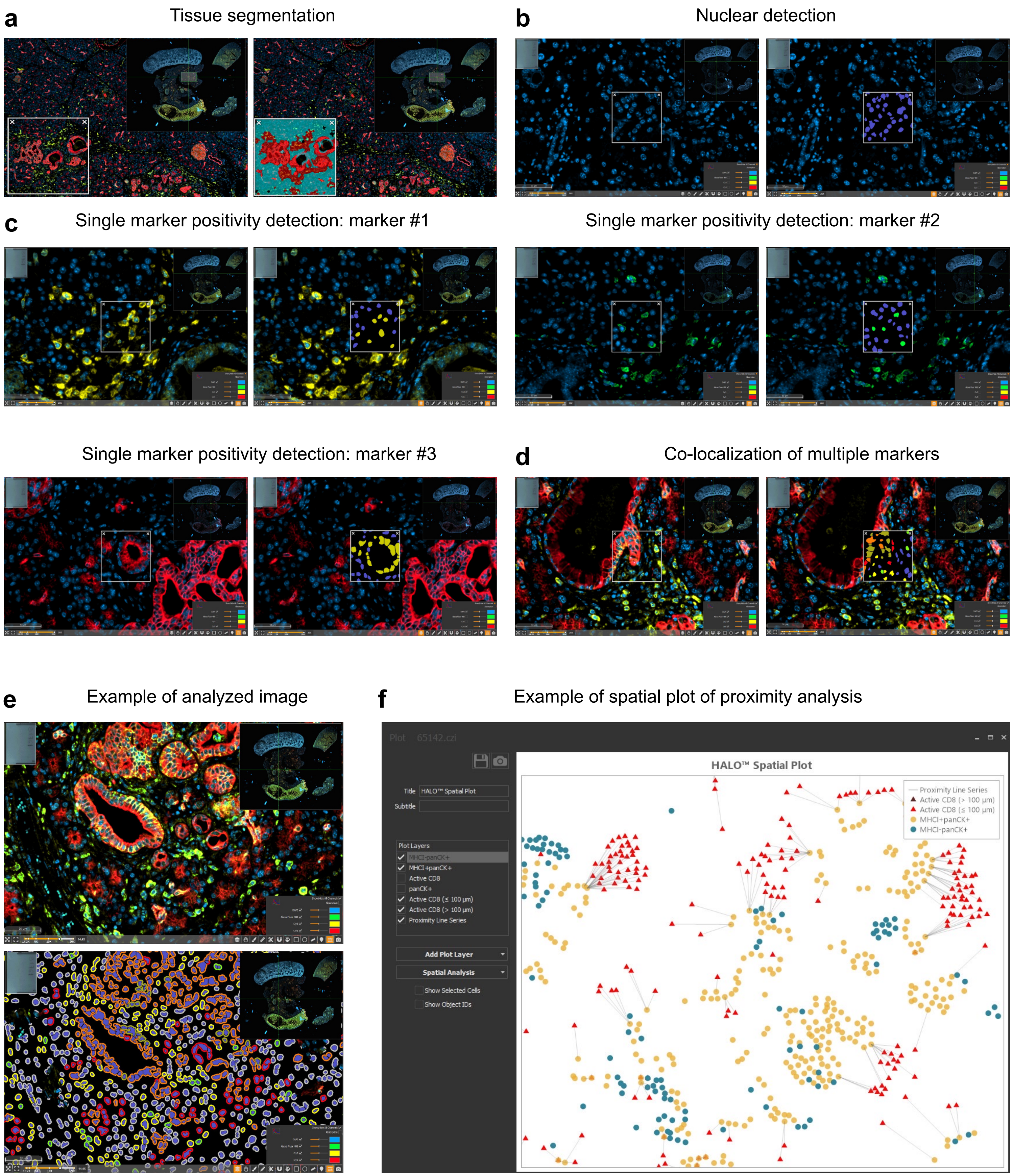

Supplementary Figure 12

Quantification of mIF staining by HALO. (a) Tissue segmentation to define tumor (red), stroma (green), and empty space (black). (b) Nuclear detection to assess the cell number in each slide. (c) Definition of positivity of individual markers. (d) Co-localization of multiple markers. (e) Example of analyzed image as visualized by co-expression of multiple markers. (n=12 for untreated *CKP* tumors harvested at different developmental stages). Representative images are shown. (f) Example of spatial plot of proximity analysis demonstrating the target cells ($CD8^+$ $GzmB^+$, Active CD8) within 100um of $MHCI^+panCK^+$ cells.

Supplementary Tables: S1-7

Supplementary Table 1. Association of PGRN level with clinicopathological parameters and immune markers in CONKO-001 cohort

	PGRN_Lo	PGRN_Hi	*P* value
Tumor size_2 classes			
T1-2	6	1	*0.107*
T3-4	30	34	
Ki67			
<50	17	9	*0.067*
50+	16	22	
SMA_intensity			
Low	2	7	*0.247*
High	16	21	
P53 intensity			
0-1	13	9	*0.292*
2-3	19	23	
$CD8^+$			
<42	9	16	*0.037*
42+	25	15	
Stromal $CD8^+$			
≤25	8	12	*0.185*
25+	26	19	
$CD103^+$			
≤3	27	23	*0.251*
3+	6	10	

Supplementary Table 2. Association of PGRN level with KRAS mutational status in CONKO-001 cohort

	PGRN_Lo	PGRN_Hi	*P* value
PGRN (PanCK$^+$Tumor cells)			
Wild-type	5	5	*0.426*
Mutated	13	23	
PGRN (CD68$^+$ Macrophages)			
Wild-type	5	5	*0.876*
Mutated	19	17	
PGRN (Total)			
Wild-type	5	5	*0.638*
Mutated	15	21	

Supplementary Table 3. IHC antibodies

Antigen	Clone	Manufacturer	Dilution	Reactivity
PGRN	A23	Ref [1]	1:200	Human, Mouse
PanCK	PCK-26	Abcam	1:100	Human, Mouse
Ki67	Polyclonal	Abcam	1:200	Mouse
F4/80	BM8	BMA biomedicals	1:200	Mouse
MRC1	Polyclonal	Abcam	1:200	Mouse
INOS	Polyclonal	Abcam	1:200	Mouse
Phospho-STAT1	M135	Abcam	1:100	Mouse
Foxp3	FJK-16s	ThermoFisher	1:50	Mouse
Cl. caspase 3	5A1E	Cell Signaling	1:100	Mouse
MHC I (H-2Db)	AF6-88.5.5.3	ThermoFisher	1:100	Mouse
MHC II	M5/114.15.2	ThermoFisher	1:100	Mouse
MHCI (HLA-A)	C-6	Santa Cruz	1:100	Human
CD3	Polyclonal	Abcam	1:100	Mouse
CD4	RM4-5	BD	1:50	
CD8	SP16	Abcam	1:100	Human
CD8	EPR20305	Abcam	1:100	Mouse
T-bet	4B10	eBioscience	1:50	Mouse
Eomes	Dan11mag	eBioscience	1:50	Mouse
Granzyme B	Polyclonal	Abcam	1:500 (hu), 1:100 (ms)	Human, Mouse
α-sma	Polyclonal	Abcam	1:100	Mouse
Lamp1	Polyclonal	Abcam	1:200	Mouse
LC3B	Polyclonal	Abcam	1:100	Mouse
CD68	KP1	Abcam	1:400	Human

Supplementary Table 4. Sequential multiplexed immunofluorescence staining protocol

Antigen	Primary antibody			TSA fluorophore	
	Clone	Manufacturer	Dilution	Fluorophore	Dilution
Fig. 1, 4					
CD68	KP1	Abcam	1:400	Opal520	1:200
PGRN	A23	Ref [1]	1:200	Opal570	1:200
PanCK	PCK-26	Abcam	1:100	Opal780	1:100
Fig. 2					
PGRN	A23	Ref [1]	1:200	Opal570	1:200
CD8	SP16	Abcam	1:100	Opal480	1:200
GzmB	Polyclonal	Abcam	1:500	Opal690	1:200
MHCI (HLA-A)	C-6	Santa Cruz	1:100	Opal520	1:200
PanCK	PCK-26	Abcam	1:100	Opal780	1:100
Fig. 5					
CD8	SP16	Abcam	1:100	Opal650	1:200
GzmB	Polyclonal	Abcam	1:500	Opal520	1:200
T-bet	4B10	eBioscience	1:50	Opal570	1:200
Fig. 6					
PGRN	A23	Ref [1]	1:200	Opal570	1:200
CD8	EPR20305	Abcam	1:100	Opal480	1:200
MHCI (H2Db)	AF6-88.5.5.3	Thermofisher	1:100	Opal690	1:200
PanCK	PCK-26	Abcam	1:100	Opal780	1:100
Fig. S5					
PDPN	Polyclonal	Abcam	1:250	Opal520	1:200
Ly6C	ER-MP20	Abcam	1:100	Opal620	1:200

Supplementary Table 5a. List of mouse strains

Gene	Genetic	Strain	References
$Kras^{fl}$	*LSL-KrasG12D* knock-in	$Kras^{tm4Tyj}$	[2]
$Ptf1a^{Cre}$	*Cre* knock-in	$Ptf1a^{tm1(cre)Hnak}$	[3]
$Trp53^{fl}$	*LoxP*-sites knock-in	$Trp53^{tm1Brn}$	[4]
$Kras^{frt}$	*FSF-KrasG12D* knock-in	$Kras^{tm1Dsa}$	[5]
$Ptf1a^{Flp}$	*Flp* knock-in	$Ptf1a^{tm(flp)}$	unpublished
$Trp53^{frt}$	*frt*-sites knock-in	$Trp53^{tm1.1Dgk}$	[6]
Rosa26 locus (Cre^{ERT2})	*FSF-*Cre^{ERT2} *knock-in*	$Gt(ROSA)26Sor^{tm3(CAG\text{-}Cre/ERT2)Das}$	[5]
Rosa26 locus (GP^{fl})	*LSL-GP knock-in*	$Gt(ROSA)26Sor^{tmloxP\text{-}STOP\text{-}loxP\text{-}GP\text{-}IRES\text{-}YFP}$	[7]
unknown locus	*TCR transgene*	$Tg(TcrLCMV)^{327Sdz}$	[8]

Supplementary Table 5b. Interbred mouse strains and description.

Strains intercrossed	Strain (abbreviated)	Action	Genotype
Ptf1a$^{tm1(cre)Hnak}$ *Kras^{Tm4Tyj}* *Trp53^{tm1Brn}*	*CKP*	Induction of spontaneous PDAC through CRE-induced pancreas-specific activation of mutant KRAS^{G12D} and homozygous loss of Tp53	*Ptf1a$^{wt/Cre}$;Kras$^{wt/LSL-G12D}$;p53$^{fl/fl}$*
Kras^{tm1Dsa} *Ptf1a$^{tm(flp)}$* *Trp53$^{tm1.1Dgk}$* *Gt(ROSA)26Sor$^{tm3(CAG-Cre/ERT2)Das}$* *Gt(ROSA)26Sor$^{tmloxP-STOP-loxP-GP-IRES-YFP}$*	*FKPC2GP*	Induction of spontaneous PDAC through Flp-induced pancreas-specific activation of mutant KRAS^{G12D} and homozygous loss of Tp53. Tamoxifen-inducible pancreas-specific expression of YFP and glycoprotein	*Ptf1a$^{wt/Flp}$;Kras$^{wt/FSF-G12D}$;p53$^{frt/frt}$;ROSA26$^{FSF-CreERT2/LSL-GP}$*

Supplementary Table 6. FACS antibodies

Antigen	Conjugate	Clone	Manufacturer	Isotype	Amount/test or dilution
PGRN	Unconjugated	A23	Ref [1]	Mouse IgG1k	1ug
HLA-A/B/C	FITC	W6/32	Biolegend	Mouse IgG2a	1:20
HLA-DR	FITC	L243	Biolegend	Mouse IgG2a	2ug
LCMV-gp33	FITC	--	In house	--	1:100
H2Db	FITC	KH95	Biolegend	Mouse IgG2b	1ug
CD3	APC	145-2C11	BD	Armenian Hamster IgG1k	1:50
CD45.1	FITC	A20	Thermofisher	Mouse IgG2a	0.5ug
CD8	eFluor450	53-6.7	ebiosciences	Rat Ig2a	0.25
GzmB	Alexa Fluor 647	GB11	Biolegend	Mouse IgG1k	1:100
TNF	PE	MP6-XT22	Thermofisher	Rat IgG1k	1:100
IFNg	PE	XMG1.2	Thermofisher	Rat IgG1k	0.25
EpCAM	APC	G8.8	Thermofisher	Rat IgG2ak	0.125
α-sma	Unconjugated	1A4	Thermofisher	Mouse IgG2a	0.5
Podoplanin	Alexa Fluor488	8.1.1	Biolegend	Syrian Hamster IgG	0.25
Ly6C	APC	HK1.4	Biolegend	Rat IgG2cκ	0.25
MHCII	Unconjugated	M5/114/15/2	Thermofisher	Rat IgG2bk	0.125
PDGFR1	PE	APA5	Biolegend	Rat IgG2ak	0.25

Supplementary Table 7. Immunofluorescence staining and antibodies

Primary antibodies	Clone	Manufacturer	Dil.	Secondary antibodies	Manufacturer	Dil.
LC3B	Poly	Abcam	1:500	Dylight594-Goat anti-rabbit	Thermofisher	1:500
HLA-A/B/C	W6/32	Biolegend	1:150	Alexa Fluor488-Goat anti-mouse	Thermofisher	1:500
H2Db	AF6-88.5.5.3	ThermoFisher	1:150	Alexa Fluor488-Goat anti-mouse	Thermofisher	1:500
Rab7	D95F2	Cell signaling	1:100	Dylight594-Goat anti-rabbit	Thermofisher	1:500
Lamp1	H4A3	Abcam	1:200	Alexa Fluor488-Goat anti-mouse	Thermofisher	1:500

Reference

1. Ho JC, Ip YC, Cheung ST, Lee YT, Chan KF, Wong SY, Fan ST: Granulin-epithelin precursor as a therapeutic target for hepatocellular carcinoma. *Hepatology* 2008, 47(5):1524-1532.
2. Jackson EL, Willis N, Mercer K, Bronson RT, Crowley D, Montoya R, Jacks T, Tuveson DA: Analysis of lung tumor initiation and progression using conditional expression of oncogenic K-ras. *Genes & development* 2001, 15(24):3243-3248.
3. Nakhai H, Sel S, Favor J, Mendoza-Torres L, Paulsen F, Duncker GI, Schmid RM: Ptf1a is essential for the differentiation of GABAergic and glycinergic amacrine cells and horizontal cells in the mouse retina. *Development* 2007, 134(6):1151-1160.
4. Marino S, Vooijs M, van Der Gulden H, Jonkers J, Berns A: Induction of medulloblastomas in p53-null mutant mice by somatic inactivation of Rb in the external granular layer cells of the cerebellum. *Genes & development* 2000, 14(8):994-1004.
5. Schonhuber N, Seidler B, Schuck K, Veltkamp C, Schachtler C, Zukowska M, Eser S, Feyerabend TB, Paul MC, Eser P *et al*: A next-generation dual-recombinase system for time- and host-specific targeting of pancreatic cancer. *Nature medicine* 2014, 20(11):1340-1347.
6. Lee CL, Moding EJ, Cuneo KC, Li Y, Sullivan JM, Mao L, Washington I, Jeffords LB, Rodrigues RC, Ma Y *et al*: p53 functions in endothelial cells to prevent radiation-induced myocardial injury in mice. *Sci Signal* 2012, 5(234):ra52.
7. Page N, Klimek B, De Roo M, Steinbach K, Soldati H, Lemeille S, Wagner I, Kreutzfeldt M, Di Liberto G, Vincenti I *et al*: Expression of the DNA-Binding Factor TOX Promotes the Encephalitogenic Potential of Microbe-Induced Autoreactive CD8(+) T Cells. *Immunity* 2018, 48(5):937-950 e938.
8. Pircher H, Burki K, Lang R, Hengartner H, Zinkernagel RM: Tolerance induction in double specific T-cell receptor transgenic mice varies with antigen. *Nature* 1989, 342(6249):559-561.
