# Supplementary material for: Progranulin mediates immune evasion of pancreatic ductal adenocarcinoma through regulation of MHCI expression
Source: Nat Commun. 2022 Jan 10;13:156. doi: 10.1038/s41467-021-27088-9 (PMC8748938; doi:10.1038/s41467-021-27088-9)
Supplement: Supplementary file 3 — Description of Additional Supplementary Files [file 41467_2021_27088_MOESM3_ESM.pdf]

Description of Additional Supplementary Files

File Name: Supplementary Data 1
Description: Stromal cell type estimation in Maurer et al dataset (GSE93326)

File Name: Supplementary Data 2
Description: Geneset enrichment analysis in Maurer et al dataset (GSE93326)

File Name: Supplementary Data 3
Description: Differentially expressed gene analysis in Maurer et al dataset (GSE93326)
