# Supplementary material for: Progranulin mediates immune evasion of pancreatic ductal adenocarcinoma through regulation of MHCI expression
Source: Nat Commun. 2022 Jan 10;13:156. doi: 10.1038/s41467-021-27088-9 (PMC8748938; doi:10.1038/s41467-021-27088-9)
Supplement: Supplementary file 7 — Reporting Summary [file 41467_2021_27088_MOESM7_ESM.pdf]

nature research

Corresponding author(s): Jens T Siveke

Last updated by author(s): Sep 15, 2021

Reporting Summary

Nature Research wishes to improve the reproducibility of the work that we publish. This form provides structure for consistency and transparency in reporting. For further information on Nature Research policies, see our Editorial Policies and the Editorial Policy Checklist.

Statistics

For all statistical analyses, confirm that the following items are present in the figure legend, table legend, main text, or Methods section.

n/a	Confirmed	
☐	☒	The exact sample size (*n*) for each experimental group/condition, given as a discrete number and unit of measurement
☐	☒	A statement on whether measurements were taken from distinct samples or whether the same sample was measured repeatedly
☐	☒	The statistical test(s) used AND whether they are one- or two-sided<br>*Only common tests should be described solely by name; describe more complex techniques in the Methods section.*
☒	☐	A description of all covariates tested
☐	☒	A description of any assumptions or corrections, such as tests of normality and adjustment for multiple comparisons
☐	☒	A full description of the statistical parameters including central tendency (e.g. means) or other basic estimates (e.g. regression coefficient) AND variation (e.g. standard deviation) or associated estimates of uncertainty (e.g. confidence intervals)
☐	☒	For null hypothesis testing, the test statistic (e.g. *F*, *t*, *r*) with confidence intervals, effect sizes, degrees of freedom and *P* value noted<br>*Give P values as exact values whenever suitable.*
☒	☐	For Bayesian analysis, information on the choice of priors and Markov chain Monte Carlo settings
☒	☐	For hierarchical and complex designs, identification of the appropriate level for tests and full reporting of outcomes
☐	☒	Estimates of effect sizes (e.g. Cohen's *d*, Pearson's *r*), indicating how they were calculated

Our web collection on statistics for biologists contains articles on many of the points above.

Software and code

Policy information about availability of computer code

Data collection	Flow cytometry: FACSCelesta (BD); Microscopy: Axio Scanner Z.1 (Zeiss)
Data analysis	Microscopic images: Definiens Tissue Studio (ver 2.6), HALO (ver 3.2), ZEISS ZEN 2.3, Imaging Software.ImageJ (ver 2.0.0) and R Package Spatstat (ver.1.59.0), and HALO (ver 3.2) for spatial image analysis; Flow cytometry: FACS Diva v.5, FlowJo ver 7.5.5; Transcriptional profiling of dataset from Maurer et al (GSE93326): EdgeR (3.28.0), fgsea (1.12.0); cell type estimation based on transcriptome of dataset from Maurer et al (GSE93326): R environment (3.6.1); Other statistical analyses: GraphPad Prism 6, SPSS ver 22.

For manuscripts utilizing custom algorithms or software that are central to the research but not yet described in published literature, software must be made available to editors and reviewers. We strongly encourage code deposition in a community repository (e.g. GitHub). See the Nature Research guidelines for submitting code & software for further information.

Data

Policy information about availability of data

All manuscripts must include a data availability statement. This statement should provide the following information, where applicable:

- Accession codes, unique identifiers, or web links for publicly available datasets
- A list of figures that have associated raw data
- A description of any restrictions on data availability

Source data are provided with this paper. The data used to analyze for transcriptomic profiling of GRN high and low tumor epithelium and stroma is available in Gene Expression Omnibus (GEO) database under accession code GSE93326.

Field-specific reporting

Please select the one below that is the best fit for your research. If you are not sure, read the appropriate sections before making your selection.

☒ Life sciences ☐ Behavioural & social sciences ☐ Ecological, evolutionary & environmental sciences

For a reference copy of the document with all sections, see nature.com/documents/nr-reporting-summary-flat.pdf

Life sciences study design

All studies must disclose on these points even when the disclosure is negative.

Sample size

For studying PGRN expression in human PDAC, sample sizes are 54, 31 and 71 patients from Essen cohort, Nijmegen cohort, and CONKO-001, respectively. No sample size calculation was performed as sample sizes were determined based on the sample availability of these retrospective studies. All patients collected with FFPE samples were used. Sample sizes of Essen cohort and CONKO-001 cohorts were considered adequate based on comparisons with data reported from similar studies in the field that used similar sample sizes. Nijmegen cohort has relatively small sample size and therefore statistical significance was not reached. However, this cohort only served as a validation cohort where we could observe the same trend as observed in the other two cohorts to support our hypothesis.
For in vivo PGRN blockade during early PDAC development (treatment period: 2 weeks), 8 mice per treatment group (PGRN antibody or mIg control) were included. Sample size was calculated by power analysis.

Data exclusions

No data was excluded from the analyses of this study. None of the mice with the appropriate genotype was excluded.

Replication

For human cohorts, IHC stainings were performed on FFPE samples of all patients included in the cohorts (54, 31 and 71 patients from Essen cohort, Nijmegen cohort, and CONKO-001, respectively). For multiplex immunofluorescence (mIF) staining in human PDAC, samples from 8 patients were stained and analysed.
For mouse studies, IHC staining was performed on all animals included in the experiments (untreated controls: n=5; mIg: n=8; PGRN Ab: n=8; aCD8: n=7; PGRN Ab+aCD8: n=6; GP+mIg: n=4; GP+PGRN Ab:n=4). mIF stainings were performed on 4 samples (for descriptive characterization) or all treated animals (n=8, for statistical analysis). All stainings were quantified and analyzed by software. Quantified results were all shown in the manuscript.
All in vitro assays were repeated at least 4 times.
All attempts at replication were successful.

Randomization

All sample allocation was random in the study.

Blinding

The investigators were blinded to group allocation during data collection and analysis.

Reporting for specific materials, systems and methods

We require information from authors about some types of materials, experimental systems and methods used in many studies. Here, indicate whether each material, system or method listed is relevant to your study. If you are not sure if a list item applies to your research, read the appropriate section before selecting a response.

Materials & experimental systems

n/a	Involved in the study	
☐	☒	Antibodies
☐	☒	Eukaryotic cell lines
☒	☐	Palaeontology and archaeology
☐	☒	Animals and other organisms
☐	☒	Human research participants
☒	☐	Clinical data
☒	☐	Dual use research of concern

Methods

n/a	Involved in the study	
☒	☐	ChIP-seq
☐	☒	Flow cytometry
☒	☐	MRI-based neuroimaging

Antibodies

Antibodies used

Immunohistochemistry:
PGRN, Clone: A23, provided by co-author Cheung ST (Ref [1]); PanCK, Clone: PCK-26, Manufacturer: Abcam, ab6401; Ki67, Clone: Polyclonal, Manufacturer: Abcam, ab15580; F4/80, Clone: BM8, Manufacturer: BMA biomedicals, T-2028; MRC1, Clone: Polyclonal, Manufacturer: Abcam, ab64693; INOS, Clone: Polyclonal, Manufacturer: Abcam, ab115819; Phospho-STAT1, Clone: M135, Manufacturer: Abcam, ab29045;Foxp3, Clone: FJK-16s, Manufacturer: ThermoFisher, 14-5773-82; Cl. caspase 3, Clone: 5A1E, Manufacturer: Cell Signaling, 9664S; MHC I (H-2Db), Clone: AF6-88.5.5.3, Manufacturer: ThermoFisher, 13-5958-82; MHC II, Clone: M5/114.15.2, Manufacturer: ThermoFisher, 14-5321-82; MHCI (HLA-A), Clone: C-6, Manufacturer: Santa Cruz, sc-365486; CD3, Clone: Polyclonal, Manufacturer: Abcam, ab16669; CD4, Clone: RM4-5, Manufacturer: BD, 550280; CD8, Clone: SP16, Manufacturer: Abcam, ab101500; CD8, Clone: EPR20305, Manufacturer: Abcam, ab209775; T-bet, Clone: 4B10, Manufacturer: eBioscience, 14-5825-82; Eomes, Clone: Dan11mag, Manufacturer: eBioscience, 14-4875-82; Granzyme B, Clone: Polyclonal, Manufacturer:

Abcam, ab4059;α-sma, Clone: Polyclonal, Manufacturer: Abcam, ab5694; Lamp1, Clone: Polyclonal, Manufacturer: Abcam, ab24170; LC3B, Clone: Polyclonal, Manufacturer: Abcam, ab51520;CD68, Clone: KP1, Manufacturer: Abcam, ab955

Flow cytometry:
PGRN, Unconjugated, Clone: A23, Ref [1]; HLA-A/B/C, FITC, Clone: W6/32, Manufacturer: Biolegend, 311404; HLA-DR, FITC, Clone: L243, Manufacturer: Biolegend, 307604; LCMV-gp33, FITC, Clone: In house; H2Db, FITC, Clone: KH95, Manufacturer: Biolegend, 111506; CD3, APC, Clone: 145-2C11, Manufacturer: BD, 553066; CD45.1, FITC, Clone: A20, Manufacturer: Thermofisher, 11-0453-82; CD8, eFluor450, Clone: 53-6.7, Manufacturer: ebiosciences, 48-0081-82; GzmB, Alexa Fluor 647, Clone: GB11, Manufacturer: Biolegend, 515406; TNFa, PE, Clone: MP6-XT22, Manufacturer: Thermofisher, 12-7321-41; IFNg, PE, Clone: XMG1.2, Manufacturer: Thermofisher, 12-7311-41; EpCAM, APC, Clone: G8.8, Manufacturer: Thermofisher, 17-5791-82; α-sma, Unconjugated, Clone: 1A4, Manufacturer: Thermofisher, 14-9760-82; Podoplanin, AF488, Clone: 8.1.1, Manufacturer: Biolegend, 127406; Ly6C, APC, Clone: HK1.4, Manufacturer: Biolegend, 128016; MHCII, unconjugated, Clone: M5/114/15/2, Manufacturer: Thermofisher,14-5321-82; PDGFR1, PE, Clone: APA5, Manufacturer: Biolegend, 135906.

Immunocytochemistry and immunofluorescence staining
LC3B, Clone: Polyclonal, Manufacturer: Abcam, ab51520; Dylight594-Goat anti-rabbit, Manufacturer: Thermofisher, 35561; HLA-A/B/C, Clone: W6/32, Manufacturer: Biolegend, 311402; Alexa Fluor488-Goat anti-mouse, Manufacturer: Thermofisher, A28175; H2Db, Clone: AF6-88.5.5.3, Manufacturer: ThermoFisher, 13-5958-82; Alexa Fluor488-Goat anti-mouse, Manufacturer: Thermofisher, A28175; Rab7, Clone: D95F2, Manufacturer: Cell signaling, 9367S; Lamp1: Clone: H4A3; Manufacturer: Abcam, ab25630.

Ref:
1. Ho JC, Ip YC, Cheung ST, Lee YT, Chan KF, Wong SY, Fan ST: Granulin-epithelin precursor as a therapeutic target for hepatocellular carcinoma. Hepatology 2008, 47(5):1524-1532.

Validation

All antibodies used for immunohistochemistry and immunocytochemistry were validated and optimized internally based on the staining conditions and antibody dilution recommended by the manufacturers. Antibodies for flow cytometry were used at dilutions according to manufacturers' recommendation at their websites.
Immunohistochemistry:
PGRN, Clone: A23, provided and optimized by co-author Cheung ST (Ref [1]);
PanCK, Clone: PCK-26, Manufacturer: Abcam, ab6401; https://www.abcam.com/pan-Cytokeratin-antibody-PCK-26-ab6401.html
Ki67, Clone: Polyclonal, Manufacturer: Abcam, ab15580; https://www.abcam.com/Ki67-antibody-ab15580.html?gclsrc=aw.ds|aw.ds&gclid=EAIaIQobChMIus-p-cL78gIVled3Ch2EBgVHEAAYASAAEgIgPfD_BwE
F4/80, Clone: BM8, Manufacturer: BMA biomedicals, T-2028; http://www.bma.ch/en/products/t-2028
MRC1, Clone: Polyclonal, Manufacturer: Abcam, ab64693; https://www.abcam.com/mannose-receptor-antibody-ab64693.html
INOS, Clone: Polyclonal, Manufacturer: Abcam, ab115819; https://www.abcam.com/inos-antibody-sp126-ab115819.html
Phospho-STAT1, Clone: M135, Manufacturer: Abcam, ab29045; https://www.abcam.com/stat1-phospho-y701-antibody-m135-ab29045.html
Foxp3, Clone: FJK-16s, Manufacturer: ThermoFisher, 14-5773-82; https://www.thermofisher.com/antibody/product/FOXP3-Antibody-clone-FJK-16s-Monoclonal/14-5773-82
Cl. caspase 3, Clone: 5A1E, Manufacturer: Cell Signaling, 9664S; https://www.cellsignal.de/products/primary-antibodies/cleaved-caspase-3-asp175-5a1e-rabbit-mab/9664
MHC I (H-2Db), Clone: AF6-88.5.5.3, Manufacturer: ThermoFisher, 13-5958-82; https://www.thermofisher.com/antibody/product/MHC-Class-I-H-2Kb-Antibody-clone-AF6-88-5-5-3-Monoclonal/13-5958-82
MHC II, Clone: M5/114.15.2, Manufacturer: ThermoFisher, 14-5321-82; https://www.thermofisher.com/antibody/product/MHC-Class-II-I-A-I-E-Antibody-clone-M5-114-15-2-Monoclonal/14-5321-82
MHCI (HLA-A), Clone: C-6, Manufacturer: Santa Cruz, sc-365486; https://www.scbt.com/p/hnrnp-a1-antibody-f-8 maybe not the correct one
CD3, Clone: Polyclonal, Manufacturer: Abcam, ab16669; https://www.abcam.com/cd3-antibody-sp7-ab16669.html
CD4, Clone: RM4-5, Manufacturer: BD, 550280; https://www.bdbiosciences.com/en-us/products/reagents/flow-cytometry-reagents/research-reagents/single-color-antibodies-ruo/purified-rat-anti-mouse-cd4.550280
CD8, Clone: SP16, Manufacturer: Abcam, ab101500; https://www.abcam.com/cd8-alpha-antibody-sp16-ab101500.html
CD8, Clone: EPR20305, Manufacturer: Abcam, ab209775; https://www.abcam.com/cd8-alpha-antibody-epr20305-ab209775.html
T-bet, Clone: 4B10, Manufacturer: eBioscience, 14-5825-82; https://www.thermofisher.com/antibody/product/T-bet-Antibody-clone-eBio4B10-4B10-Monoclonal/14-5825-82
Eomes, Clone: Dan11mag, Manufacturer: eBioscience, 14-4875-82; https://www.thermofisher.com/antibody/product/EOMES-Antibody-clone-Dan11mag-Monoclonal/14-4875-82
Granzyme B, Clone: Polyclonal, Manufacturer: Abcam, ab4059; https://www.abcam.com/granzyme-b-antibody-ab4059.html
α-sma, Clone: Polyclonal, Manufacturer: Abcam, ab5694; https://www.abcam.com/alpha-smooth-muscle-actin-antibody-ab5694.html
Lamp1, Clone: Polyclonal, Manufacturer: Abcam, ab24170; https://www.abcam.com/lamp1-antibody-lysosome-marker-ab24170.html
LC3B, Clone: Polyclonal, Manufacturer: Abcam, ab51520; https://www.abcam.com/lc3b-antibody-ab51520.html
CD68, Clone: KP1, Manufacturer: Abcam, ab955 https://www.abcam.com/cd68-antibody-kp1-ab955.html

Flow cytometry:
PGRN, Unconjugated, Clone: A23, optimized by co-author Cheung ST, Ref [1];
HLA-A/B/C, FITC, Clone: W6/32, Manufacturer: Biolegend, 311404; https://www.biolegend.com/en-us/products/fitc-anti-human-hla-a-b-c-antibody-1871
HLA-DR, FITC, Clone: L243, Manufacturer: Biolegend, 307604; https://www.biolegend.com/en-us/products/fitc-anti-human-hla-dr-antibody-788
H2Db, FITC, Clone: KH95, Manufacturer: Biolegend, 111506; https://www.biolegend.com/en-us/products/fitc-anti-mouse-h-2d-b-antibody-325
CD3, APC, Clone: 145-2C11, Manufacturer: BD, 553066; https://www.bdbiosciences.com/en-de/products/reagents/flow-cytometry-reagents/research-reagents/single-color-antibodies-ruo/apc-hamster-anti-mouse-cd3e.553066
CD45.1, FITC, Clone: A20, Manufacturer: Thermofisher, 11-0453-82; https://www.thermofisher.com/antibody/product/CD45-1-

Antibody-clone-A20-Monoclonal/11-0453-82
CD8, eFluor450, Clone: 53-6.7, Manufacturer: ebiosciences, 48-0081-82; https://www.thermofisher.com/antibody/product/CD8a-Antibody-clone-53-6-7-Monoclonal/48-0081-82
GzmB, Alexa Fluor 647, Clone: GB11, Manufacturer: Biolegend, 515406; https://www.biolegend.com/en-us/products/alexa-fluor-647-anti-human-mouse-granzyme-b-antibody-6067
TNFa, PE, Clone: MP6-XT22, Manufacturer: Thermofisher, 12-7321-41; https://www.thermofisher.com/antibody/product/TNF-alpha-Antibody-clone-MP6-XT22-Monoclonal/12-7321-41
IFNg, PE, Clone: XMG1.2, Manufacturer: Thermofisher, 12-7311-41; https://www.thermofisher.com/antibody/product/IFN-gamma-Antibody-clone-XMG1-2-Monoclonal/12-7311-41
EpCAM, APC, Clone: G8.8, Manufacturer: Thermofisher, 17-5791-82; https://www.thermofisher.com/antibody/product/CD326-EpCAM-Antibody-clone-G8-8-Monoclonal/17-5791-82
α-sma, Unconjugated, Clone: 1A4, Manufacturer: Thermofisher, 14-9760-82; https://www.thermofisher.com/antibody/product/Alpha-Smooth-Muscle-Actin-Antibody-clone-1A4-Monoclonal/14-9760-82
Podoplanin, AF488, Clone: 8.1.1, Manufacturer: Biolegend, 127406; https://www.biolegend.com/en-us/products/alexa-fluor-488-anti-mouse-podoplanin-antibody-4751
Ly6C, APC, Clone: HK1.4, Manufacturer: Biolegend, 128016; https://www.biolegend.com/en-us/products/apc-anti-mouse-ly-6c-antibody-6047
MHCII, unconjugated, Clone: M5/114/15/2, Manufacturer: Thermofisher,14-5321-82; https://www.thermofisher.com/antibody/product/MHC-Class-II-I-A-I-E-Antibody-clone-M5-114-15-2-Monoclonal/14-5321-82
PDGFR1, PE, Clone: APA5, Manufacturer: Biolegend, 135906; https://www.biolegend.com/en-us/products/pe-anti-mouse-cd140a-antibody-6253

Immunocytochemistry and immunofluorescence staining
LC3B, Clone: Polyclonal, Manufacturer: Abcam, ab51520; https://www.abcam.com/lc3b-antibody-ab51520.html
Dylight594-Goat anti-rabbit, Manufacturer: Thermofisher, 35561; https://www.thermofisher.com/antibody/product/Goat-anti-Rabbit-IgG-H-L-Cross-Adsorbed-Secondary-Antibody-Polyclonal/35561
HLA-A/B/C, Clone: W6/32, Manufacturer: Biolegend, 311402; https://www.biolegend.com/en-us/products/purified-anti-human-hla-a-b-c-antibody-1874
Alexa Fluor488-Goat anti-mouse, Manufacturer: Thermofisher, A28175; https://www.thermofisher.com/antibody/product/Goat-anti-Mouse-IgG-H-L-Secondary-Antibody-Recombinant-Polyclonal/A28175
H2Kb, Clone: AF6-88.5.5.3, Manufacturer: ThermoFisher, 13-5958-82; https://www.thermofisher.com/antibody/product/MHC-Class-I-H-2Kb-Antibody-clone-AF6-88-5-5-3-Monoclonal/13-5958-82
Alexa Fluor488-Goat anti-mouse, Manufacturer: Thermofisher, A28175; https://www.thermofisher.com/antibody/product/Goat-anti-Mouse-IgG-H-L-Secondary-Antibody-Recombinant-Polyclonal/A28175
Rab7, Clone: D95F2, Manufacturer: Cell signaling, 9367S; https://www.cellsignal.de/products/primary-antibodies/rab7-d95f2-xp-rabbit-mab/9367?site-search-type=Products&N=4294956287&Ntt=9367s&fromPage=plp&_requestid=1239931
Lamp1: Clone: H4A3; Manufacturer: Abcam, ab25630. https://www.abcam.com/lamp1-antibody-h4a3-ab25630.html

Eukaryotic cell lines

Policy information about cell lines

Cell line source(s): Human PDAC cell lines, PaTu8988T, MiaPaCa2 and HupT4 were purchased from the American Type Culture Collection (ATCC).

Authentication: No authentication was performed.

Mycoplasma contamination: The cell lines were routinely checked for mycoplasma contamination every 2 month. All tests were negative.

Commonly misidentified lines (See ICLAC register): No commonly misidentified cell lines was used.

Animals and other organisms

Policy information about studies involving animals; ARRIVE guidelines recommended for reporting animal research

Laboratory animals: Ptf1awt/Cre;Kraswt/LSL-G12D;p53fl/fl (CKP) mice, FKPC2GP mice, Krastm4Tyj mice, Ptf1atm1(cre)Hnak mice, Trp53tm1Brn mice, Krastm1Dsa mice, Ptf1atm(flp) mice, Trp53tm1.1Dgk mice, Gt(ROSA)26Sortm3(CAG-Cre/ERT2)Das mice, Gt(ROSA)26SortmloxP-STOP-loxP-GP-IRES-YFP mice, Tg(TcrLCMV)327Sdz mice were used in the study. Both male and female, 4-6 weeks old, were used. All animals were numbered, genotypes were revealed and animals then assigned to groups for analysis. For treatment experiments mice were randomized. None of the mice with the appropriate genotype were excluded from this study. Details of original and interbred mouse strains were described in the Materials and Methods section, and supplementary table.
Mice were maintained in rooms with 12 light/12 dark cycle, 23 degree celsius, 40-60% humidity, with food and water accessible at all times.

Wild animals: No wild animals were used in the study.

Field-collected samples: No field-collected sample was used in the study.

Ethics oversight: Animal experiments were approved under license number 84-02.04.2017.A315 by the Landesamt für Natur, Umwelt und Verbraucherschutz Nordrhein-Westfalen. All animal care and protocols adhered to national (Tierschutzgesetz) and European (Directive 2010/63/EU) laws and regulations as well as European Federation of Animal Science Associations (FELASA) http://www.felasa.eu/.

Note that full information on the approval of the study protocol must also be provided in the manuscript.

Human research participants

Policy information about studies involving human research participants

Population characteristics	This is a retrospective study where the expression of PGRN was analyzed in three independent cohorts of patients from (1) the University Hospitals Essen (Essen cohort), (2) Radboud University Medical Center (Nijmegen cohort) and (3) the phase III adjuvant CONKO-001 randomized trial, Ref [1].<br>(1) For Essen cohort, clinical data were obtained from archives and electronic health records. In this exploratory retrospective study, a cohort of 54 patients (29 female, 25 male; ages range from 49 to 89) that had undergone pancreatic resection with a final histopathologic diagnosis of human PDAC between March 2006 and February 2016 was used.<br><br>(2) Additionally, 31 patient samples from Radboud University Medical Center, Nijmegen, were used to confirm the findings of Essen cohort. The Nijmegen cohort consisted of 31 patients (13 female, 18 male; ages range from 51 to 79) with histologically proven pancreatic ductal adenocarcinoma (PDAC) between November 2004 and January 2015.<br><br>(3) For CONKO-001, the clinical details of this study have been described previously trial in Ref [1]. In brief, 183 FFPE tissue samples of CONKO-001 patients were collected retrospectively. Tissues from 165 patients was suitable for tissue microarray (TMA) construction. To model the existence of intratumoral heterogeneity, three different tumor areas were selected for the construction of TMAs using a manual tissue microarrayer (Beecher Instruments, Wisconsin, USA). Here, we analyzed only the observation arm (n=71) (31 female, 40 male; ages range from 36 to 81), in order to focus on the role of PGRN in PDAC without treatment intervention.<br><br>Ref:<br>1: Oettle H, Neuhaus P, Hochhaus A, Hartmann JT, Gellert K, Ridwelski K, et al. Adjuvant chemotherapy with gemcitabine and long-term outcomes among patients with resected pancreatic cancer: the CONKO-001 randomized trial. Jama 2013;310(14):1473-81 doi 10.1001/jama.2013.279201.
Recruitment	Since this is a retrospective study, we did not participate in any patient recruitment process for all the 3 cohorts.
Ethics oversight	For Essen cohort, PDAC tumor tissue samples for biomarker staining was provided by the Essen WBE biobank following local compliant application and reporting standards as well as patient informed consent procedures approved the local ethics committee [Medical Faculty Essen-Duisburg, ref. no. 17-7340-BO (approval for PDAC biobank and correlation with clinical parameters).<br>For Nijmegen cohort, given the retrospective nature of this study and the anonymized handling of data, informed consent was waived by the medical ethical review board (region Arnhem-Nijmegen) (protocol CMO2018-4420).<br>For CONKO-001, details were previously described in Ref [1].<br><br>Ref:<br>1: Oettle H, Neuhaus P, Hochhaus A, Hartmann JT, Gellert K, Ridwelski K, et al. Adjuvant chemotherapy with gemcitabine and long-term outcomes among patients with resected pancreatic cancer: the CONKO-001 randomized trial. Jama 2013;310(14):1473-81 doi 10.1001/jama.2013.279201.

Note that full information on the approval of the study protocol must also be provided in the manuscript.

Flow Cytometry

Plots

Confirm that:

☒ The axis labels state the marker and fluorochrome used (e.g. CD4-FITC).

☒ The axis scales are clearly visible. Include numbers along axes only for bottom left plot of group (a 'group' is an analysis of identical markers).

☒ All plots are contour plots with outliers or pseudocolor plots.

☒ A numerical value for number of cells or percentage (with statistics) is provided.

Methodology

Sample preparation	For intracellular PGRN expression, cells were permeabilized with ice-cold 0.1% saponin and then incubated with antibody or equal amount of corresponding isotype control, following by FITC-goat anti-mouse antibody (BD biosciences). CD3, CD8, CD45.1 on T cells, cells were stained with corresponding antibodies or equal amount of corresponding isotype controls. For intracellular granzyme B, TNF-a and IFN-g in T cells, or LCMV-gp33 in tumor cells, cells were fixed with 4% paraformaldehyde for 10 min at 37°C. After washing twice with PBS, cells were permeabilized with 0.1% Saponin for 20 min and then stained with antibodies and corresponding isotype. Details of primary antibodies are listed in supplementary table. Cells were then washed, resuspended, and subjected to analysis.<br><br>For orthotopic transplants of GP82 cells and CKP tumors, tumors were digested into disaggregated cells, blocked with FcR block after red blood cell lysis, and then subject to subsequent staining as described above.

Instrument	FACSCelasta, BD Biosciences
Software	FACSDiva, FlowJo
Cell population abundance	For PGRN, MHC, GP and lysosome stainings of MiaPaCa2, PaTu8988T and GP82 cells, a minimum of 10,000 stained cells were analyzed for each treatment, cell viability was routinely >95%. For co-culture experiments, cytotoxicity of 10,000 GP82 cells was analyzed for each treatment; while for cytotoxic markers GzmB, TNFa and IFNg, 10,000 T cells were analyzed for each treatment. While for orthotopic transplants of GP82 cells, 10,000 of total cells disaggregated from the tumor bulk were analyzed for the abundance of infiltrating T cells and cytotoxically active T cells in each tumor.
Gating strategy	The FSC/SSC gating strategy was used to exclude cell debris and doublets.<br>For co-culture experiments, cytoxicity (PI+) of GP82 cells was analyzed on the CFSE- cells; while the abundance of cytotoxic (GzmB: Alexa Fluor 647; TNFa, IFGg: PE) CD8 (eFluor450) cells was anlyzed on the CFSE+ T cells.<br>For orthotopic transplants of GP82 cells, abundance of cytotoxic (GzmB, TNFa, IFGg: PE) cells was anlyzed on the CD3 (APC) T cells.<br>For quantification of cancer-associated fibroblasts (CAFs), myCAFs, iCAFs and apCAFs were identified by gating on PDPN+ cells, MHCII-Ly6C- population indicates myCAFs; MHCII-Ly6C+ population indicates iCAFs; while MHCII+Ly6C- population indicates apCAFs.

☒ Tick this box to confirm that a figure exemplifying the gating strategy is provided in the Supplementary Information.
